# Development of Depotentiation in Adult-Born Dentate Granule Cells

**DOI:** 10.3389/fcell.2019.00236

**Published:** 2019-10-16

**Authors:** Xiaoqing Tao, Ning Sun, Yangling Mu

**Affiliations:** ^1^Department of Physiology, School of Basic Medicine, Huazhong University of Science and Technology, Wuhan, China; ^2^Department of Neurobiology, School of Basic Medicine, Huazhong University of Science and Technology, Wuhan, China; ^3^Institute of Brain Research, Collaborative Innovation Center for Brain Science, Huazhong University of Science and Technology, Wuhan, China; ^4^Hubei Key Laboratory of Drug Target Research and Pharmacodynamic Evaluation, Huazhong University of Science and Technology, Wuhan, China

**Keywords:** LTP, LTP reversal, hippocampus, dentate granule cells, adult-born, NR2A

## Abstract

Activity-dependent synaptic plasticity, i.e., long-term potentiation (LTP), long-term depression (LTD) and LTP reversal, is generally thought to make up the cellular mechanism underlying learning and memory in the mature brain, in which *N*-methyl-D-aspartate subtype of glutamate (NMDA) receptors and neurogenesis play important roles. LTP reversal may be the mechanism of forgetting and may mediate many psychiatric disorders, such as schizophrenia, but the specific mechanisms underlying these disorders remain unclear. In addition, LTP reversal during the development of adult-born dentate granule cells (DGCs) remains unknown. We found that the expression of the NMDA receptor subunits NR2A and NR2B displayed dynamic changes during the development of postnatal individuals and the maturation of adult-born neurons and was coupled with the change in LTP reversal. The susceptibility of LTP reversal progressively increases with the rise in the expression of NR2A during the development of postnatal individual and adult-born neurons. In addition, NMDA receptor subunits NR2A, but not NR2B, mediated LTP reversal in the DGCs of the mouse hippocampus.

## Introduction

Activity-dependent synaptic plasticity is generally believed to be the cellular mechanism underlying the developmental modification of neuronal circuits (Zhang and Poo, 2001) as well as learning and memory in the animal brain (Martin et al., 2000; Malenka and Bear, 2004; Nabavi et al., 2014). Previous studies have shown that repetitive electrical stimulation of neural pathways can rapidly induce persistent changes in synaptic efficiency in the brain, such as long-term potentiation (LTP) (Bliss and Lomo, 1973; Malenka, 2003; Lynch, 2004) and long-term depression (LTD) (Collingridge et al., 2010). However, it has been recognized that LTP can be reversed by subsequent electrical activity in the CNS, namely, LTP reversal (Zhou and Poo, 2004). The LTP reversal was first described by Hesse and Teyler (1976). They demonstrated that electroconvulsive seizure activity can reverse the low frequency tetanic stimulation-induced LTP in the rat hippocampus CA1 (Hesse and Teyler, 1976). Similarly, Poo’s group found that activity-induced LTP can be quickly reversed either by spontaneous activity of subsequent tectal neurons or by exposure to random visual inputs in the developing Xenopus retinotectal system, which depend on activation of NMDARs (Zhou et al., 2003). Similarly, high frequency stimulation induces LTP in the rat hippocampus in familiar environments, which can be reversed by exploration of a novel environment (Xu et al., 1998).

NMDA receptors (NMDARs) are glutamate-gated ion channels, which are crucial for synapse structure and function in the CNS (Paoletti et al., 2013). NMDARs are heteromers (Dingledine et al., 1999) composed of two essential subunits (NR1) and two or three regulatory subunits (NR2). In the hippocampus, the regulatory subunit is usually composed of a combination of NR2A and NR2B. The NMDAR expression patterns (region, composition and level) change dynamically during postnatal development, and NR2B is gradually replaced by NR2A (Sheng et al., 1994; Loftis and Janowsky, 2003). Development-related changes may alter the functional properties of NMDARs, especially the kinetic properties of the channels (Monyer et al., 1992; Sheng et al., 1994; Takahashi et al., 1996; Flint et al., 1997; Rumbaugh and Vicini, 1999). For instance, it has been confirmed that the NR2A-containing NMDARs deactivate faster than NR2B-containing NMDARs in HEK 293 cells (Monyer et al., 1994; Vicini et al., 1998). The direction of synaptic plasticity (potentiation or depression) is controlled largely by the NMDAR deactivation kinetics and the amount of Ca^2+^ influx through NMDARs, and therefore, the composition of NR2A and NR2B in NMDARs plays a significant role in the brain (Philpot et al., 2007; Wang et al., 2011). In addition, some reports suggested that the NMDAR subunit NR2B was involved in LTD, whereas NR2A was involved in LTP reversal (Liu L. et al., 2004; Massey et al., 2004; Zhu et al., 2005).

Adult neurogenesis has been observed in different brain regions of multiple species, such as in humans and mice (van Praag et al., 2002; Boldrini et al., 2018; Pilz et al., 2018), yet its physiological significance remains essentially unknown (Schinder and Gage, 2004). Increasing evidence suggests that newborn dentate granule cells (DGCs) contribute to hippocampus-specific forms of learning and memory, which might be different from the function of mature DGCs in the adult brain (Clelland et al., 2009; Aimone et al., 2014). Adult neurogenesis in the dentate gyrus (DG) is regulated by NMDAR activation. Application of the NMDAR antagonist resulted in an increase in the number of newborn DGCs in the adult hippocampus (Cameron et al., 1995; Gould et al., 1997). In addition, it has been reported that conditional reduction in adult neurogenesis impairs LTP and LTD in the hippocampus, and the deficits in bidirectional synaptic plasticity are completely rescued when dentate gyrus neurogenesis is recovered (Massa et al., 2011). Adult-born DGCs increase the LTP amplitude and decrease the LTP induction threshold compared with mature DGCs in the adult brain (Ge et al., 2007; Dieni et al., 2016). Moreover, there are many evidences that schizophrenia animal models show impaired generation of newborn DGCs in the adult brain (Reif et al., 2006; Mao et al., 2009; Ouchi et al., 2013). A recent report indicated that Erbb4 knock-out mice show a deficit in LTP reversal, which is coupled with behavioral deficits that are thought to be the positive symptoms of schizophrenia (Shamir et al., 2012). A recent report indicated that the developmental reinforcement of LTP reversal susceptibility is associated with increased forgetting (Ge et al., 2019). However, no report has studied the reversal of LTP in adult-born DGCs, which may contribute to a better understanding of the physiological significance of adult neurogenesis and the pathologic mechanism of related schizophrenia.

We examined LTP reversal in mature and adult-born DGCs with whole-cell patch-clamp. The adult-born DGCs were labeled with retrovirus to test synaptic plasticity during their maturation. We found that LTP reversal occurred only in mature and adult-born 6-week DGCs, not in adult-born 3-week DGCs. This difference was related to the NMDAR subunit NR2A expression level elevation in adult mouse mature DGCs. Similarly, NR2A expression increased coupled with LTP reversal in adult mice during postnatal development. In conclusion, the NMDAR subunit NR2AR mediated LTP reversal in hippocampal DGCs, and NMDAR subunit NR2A expression increased with individual postnatal development and adult-born neuron maturation.

## Materials and Methods

### Retrovirus Injection

Highly concentrated murine moloney leukemia virus-based retroviral (RV) stock (6.74 × 10^8^ TU/ml) carrying the green fluorescent protein (GFP) was injected into the DG of the mouse hippocampus through stereotaxic surgery to label dividing newborn neurons. The injection site for DG was determined using spatial coordinates relative to the bregma in the mouse brain (details shown in Table 1). The mice used for RV injection were 6–7-week-old male C57BL/6 mice, who were housed in standard cages for further experimentation after surgery. All animal experimental procedures were approved by the Committee of Animal Care of Huazhong University of Science and Technology.

**TABLE 1:** Coordinates (in mm) for RV injection.

**A–P**	**M–L**	**D–V**
**(Anterior to Posterior)**	**(Medial to Lateral)**	**(Dorsal to Ventral)**
3.0	1.5	1.9
3.2	1.55	1.9
3.4	1.6	2.0
3.6	1.65	2.0
3.8	1.7	2.05
4.0	1.75	2.1

The bregma was used as a zero point to measure the anterior to posterior (A–P) and medial to lateral (M–L) coordinates. Dorsal to ventral (D–V) positions were measured from the skull at the bregma. The bregma and the lambda were on the same horizontal plane to obtain the correct coordinates. The injection quantity of the RV for each position was 0.1 μl. Images of newborn DGCs in the hippocampus with GFP^+^ were acquired on an inverted single-photon laser scanning confocal microscope (Zeiss, LSM780).

### Electrophysiology

The mice were housed in standard cages for different periods of time after RV injection that expressed GFP were anesthetized. The mouse brains were quickly removed and placed in 4°C artificial cerebrospinal fluid (ACSF) composed of (in mM) choline chloride 110, KCl 2.5, NaH_2_PO_4_ 1.25, NaHCO_3_ 26.0, CaCl_2_ 0.5, MgCl_2_ 7, D-glucose 10, Na-ascorbate 11.6, Na-pyruvate 3.1, and atropine sulfate 0.01. Horizontal slices (200 μm thick) were cut using a Leica VT1000S vibratome and then incubated in standard ACSF containing (in mM) NaCl 119, KCl 2.5, NaH_2_PO4 1.25, NaHCO_3_ 26, CaCl_2_ 2.5, MgCl_2_ 1.3, D-glucose 10 saturated with 95% O_2_ and 5% CO_2_. Hippocampus slices from the mice were kept at 35°C approximately 40 min, followed by ACSF incubation at room temperature for at least 60 min before recording. Electrophysiological recordings were performed as previously described (Mu et al., 2015). Hippocampus DGCs were visualized by an upright microscope (Olympus, BX51WI) with infrared differential interference contrast (DIC) optics. The newborn DGCs from RV-injected mice were visually identified by their GFP and neuronal morphology, usually located in the inner DGC layer. The excitatory postsynaptic potentials (EPSPs) of mature DGCs (GFP^–^) located in the outer DGC layer (Kuhn et al., 1996; Schmidt-Hieber et al., 2004; Esposito et al., 2005) in the brain slice were recorded. The slice was perfused with oxygen-saturated ACSF containing 50 μM picrotoxin to block GABAergic synaptic transmission during recording at room temperature in the chamber. The micropipettes (3–5 ΩM) were tip-filled with internal solution composed of (in mM) K-gluconate 128, KCl 17.5, NaCl 9, MgCl_2_ 1, EGTA 0.2, and HEPES 10 (pH 7.4) and back-filled with the same internal solution containing amphotericin B (200 μg/ml) to perform the whole-cell perforated patch recordings for recording the EPSPs. A bipolar electrode (World Precision Instruments) was placed in the stratum molecular to stimulate the medial perforant pathway input to the dentate gyrus in the hippocampus slice. DGCs were held at −70 mV to record the EPSPs in current-clamp mode. For characterization of excitatory postsynaptic currents (EPSCs), potassium salt was substituted with cesium salt in the intracellular solution, which was composed of (in mM) Cs-methanesulfonate 128, CsCl 17.5, NaCl 9, MgCl_2_ 1, EGTA 0.2, and HEPES 10 (pH 7.4). α-Amino-3-hydroxy-5-methyl-4-isoxazole-propionic acid receptor (AMPAR)-mediated EPSC recorded at −70 mV in whole-cell voltage-clamp with picrotoxin (PTX, 50 μM), NMDAR-mediated EPSC recorded in the presence of CNQX (10 μM) and PTX at +40 mV in current-clamp mode. Deactivation decay times of averaged EPSCs from 10 continuous EPSC sweeps were derived from fitting to double exponential equations, $T\,\left(t\right)=A\,1\times e^{\left(-\frac{t}{t\,a\,u\,1}\right)}+A\,2\times e^{\left(-\frac{t}{t\,a\,u\,2}\right)}$, where *A1* and *A2* are the amplitudes of the fast and slow decay components, and *tau1* and *tau2* are their respective decay time constants used to fit the data. A weighted mean decay time constant was used to compare decay times: $t\,a\,u=\left(\frac{A\,1}{A\,1+A\,2}\right)\times t\,a\,u\,1+\left(\frac{A\,2}{A\,1+A\,2}\right)\times t\,a\,u\,2$ (Rumbaugh and Vicini, 1999). The antagonists for NMDAR subunits were added to the ACSF. All drugs were purchased from Sigma.

Data were collected using a Multiclamp 700B computer-controlled current and voltage clamp (Molecular Devices) and acquired with a Digidata 1550B low-noise data acquisition system (Molecular Devices) at 5 kHz. The input resistance was obtained from hyperpolarizing current injections of 5 pA into the DGCs, which were monitored continuously during recordings. The series resistance was controlled below 20 MΩ. Data were accepted for analysis only if both series and input resistances remained relatively constant (<20% change) throughout the experiment. LTP was induced with five episodes of theta-burst stimulation (TBS) applied at 0.1 Hz. Each episode of TBS consisted of 10 trains of stimuli delivered every 200 ms, with ten pulses at 100 Hz in each train (Supplementary Figure S1). Reversal LTP was induced with a combination of presynaptic stimulation and postsynaptic spikes, in which the presynaptic stimulation was 70 ms anterior to the postsynaptic spikes. The presynaptic stimulation and the postsynaptic spikes both consisted of 120 trains at 0.2 Hz, and each train contained 5 pulses at 20 Hz (Supplementary Figure S1). The extent of LTP was quantified by averaging the amplitude of EPSPs during the last 10 min of experiments and normalizing the result to the mean baseline value. Data were compared with either Student’s *t*-test (paired or unpaired), and results associated with *p* < 0.05 were considered statistically significant.

### DGC-Specific and DG-Specific Real-Time PCR

Bilateral DGs were separated from the hippocampus under a stereomicroscope to extract the tissue RNA of DGs. The RNA was converted to cDNA through reverse transcription (Vazyme, R223) according to the operation manual for real-time PCR. Entire DGCs were harvested from acute mouse brain slices by patch-clamp capillaries with a tip diameter of approximately 5 μm (Song et al., 2016). A total of 20 cells were collected into one pipette to increase the quantity of RNA, and the pipette contents were then moved into a sterile 0.5 ml PCR tube containing TRK buffer (TakaRa, RR047Q) to extract the neurons RNA of DGCs. The RNA was converted to cDNA through reverse transcription (OMEGA, R6831) according to the product instructions. The DG and DGC cDNA samples were subsequently analyzed by forty rounds of PCR amplification (Vazyme, Q311) using specific primers for Grin2A (NR2A), Grin2B (NR2B) and the housekeeping gene GAPDH (Table 2). GAPDH could be detected reliably from all samples tested. Real-time PCR experiments were performed in triplicate, and each sample was normalized to the control housekeeping gene GAPDH. Electrophoresis images were obtained using the gel imaging analysis system to verify the product of NR2A, NR2B and GAPDH after PCR amplification.

**TABLE 2:** Primer sequences used for real-time PCR analysis.

**Gene**	**Forward sense primer**	**Reverse antisense primer**
NR2A	GTTTGTTGGTGACGGTGAGA	AAGAGGTGCTCCCAGATGAA
NR2B	ATGTGGATTGGGAGGATAGG	TCGGGCTTTGAGGATACTTG
GAPDH	TCTCCTGCGACTTCAACA	TGTAGCCGTATTCATTGTCA

### Statistical Analysis

Statistical analyses were performed using two-tailed Student’s *t* tests (paired or unpaired). Data are expressed as the mean ± SEM. A *p*-value less than 0.05 was considered statistically significant.

## Results

### Characteristics of LTP and LTP Reversal in the DGCs of the Adult Mouse Hippocampus

**FIGURE 1:**
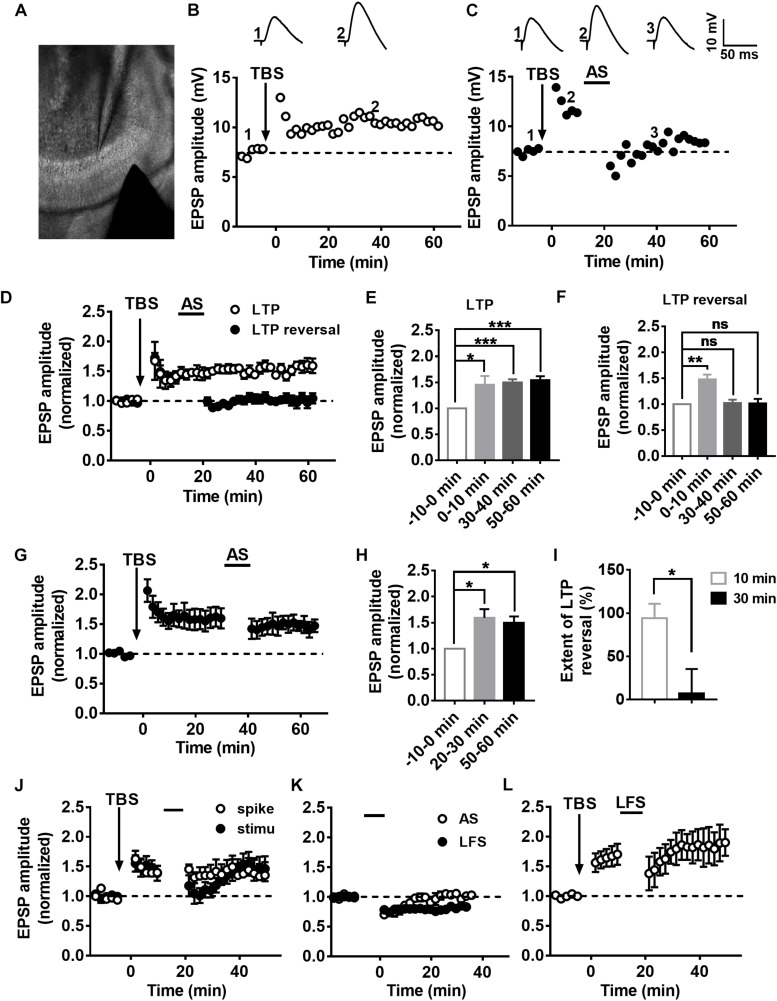
Characteristics of LTP and LTP reversal in the DGCs of the adult mouse hippocampus. **(A)** A photograph of the EPSP recording setup by whole-cell patch-clamp in adult mouse hippocampal DGCs. **(A,B)** LTP **(B)** and LTP reversal **(C)** were recorded from mature DGCs at the adult brain. Shown in the top row are typical example traces of LTP of EPSPs recorded under the whole-cell current-clamp. Representative EPSPs were taken before and after LTP induction by a physiological relevant TBS (arrow) at specific time points (1 and 2) or after LTP reversal by a combination of presynaptic stimulation and postsynaptic spikes (AS, line) at the time point (3) indicated in the graph. The AS was delivered 10 min after LTP induction, which effectively reversed the potentiation in adult brain slices. **(D)** Summary of LTP (open circles) and LTP reversal (solid circles) recorded from DGCs in the adult hippocampal slices. Normalized EPSP amplitudes are shown in **D**. **(E,F)** Summary of means of the different time points in LTP (baseline: –10–0 min, LTP: 0–10 min, 30–40 min, 50–60 min) and the LTP reversal (baseline: –10–0 min, LTP: 0–10 min, LTP reversal: 30–40 min, 50–60 min) recordings. Values represent the mean ± SEM (^∗^*p* < 0.05, ^∗∗^*p* < 0.01, ^∗∗∗^*p* < 0.001; paired *t*-test). **(G,H)** The AS failed to reverse LTP after 30 min from LTP induction. Values represent the mean ± SEM (^∗^*p* < 0.05; paired *t*-test). **(I)** Summary of the time window for the effectively reversing LTP by AS from **(D,G)**. Values represent the mean ± SEM (^∗^*p* < 0.05; unpaired *t*-test). **(J)** Induction of LTP reversal was failed by either presynaptic stimulation (solid circles) or postsynaptic spikes (open circles) alone. **(K)** The effect of AS (open circles) and low frequency stimulation (LFS, 1 Hz, solid circles) on the basic synaptic plasticity. The AS transiently suppressed basal synaptic transmission. However, 1 Hz LFS led to stable LTD in the hippocampal DGCs. **(L)** LTP reversal failed by 1 Hz LFS after 10 min from LTP induction.

To evaluate the LTP and LTP reversal, EPSPs of DGCs were recorded in current-clamp mode with low-frequency stimulation (20 s per interval) of the medial perforant pathway (Figure 1A). To examine the synaptic plasticity of DGCs, we used a TBS paradigm (Supplementary Figure S1) that mimics the physiological firing pattern commonly observed in hippocampal DGCs *in vivo* (Skaggs et al., 1996) to induce LTP (Larson and Lynch, 1986). Figures 1B,D illustrates a typical LTP of an EPSP recorded from DGCs with TBS stimulation. Here, we calculate the normalized EPSP amplitude by averaging the baseline as the standardized reference value, and we set the time point after applying the TBS as 0 min for all LTP and LTP reversal cartograms unless otherwise stated. During the recordings, LTP was maintained at a stable level for more than 60 min (Figure 1E, paired *t*-test, compared with the baseline, 0–10 min: 1.45 ± 0.1715, *P* = 0.0468; 30–40 min: 1.498 ± 0.062, *P* = 0.0005; 50–60 min: 1.545 ± 0.07196, *P* = 0.0006; *n* = 6 cells). After 10 min of LTP induction, an associated 10-min stimulus of presynaptic stimulation and postsynaptic spikes (associated stimulation, AS, Supplementary Figure S1) that simulates the spontaneous activity and firing pattern of Xenopus tectal neurons exposed to a random visual input which can reverse the LTP *in vitro* (Zhou et al., 2003) was applied to reverse the LTP (Figures 1C,D). LTP (open circles, *n* = 6 cells) and LTP reversal (solid circles, *n* = 7 cells) were successfully induced after applying the relevant stimuli to the hippocampus (Figure 1D). The LTP amplitude was efficiently reversed almost to the baseline level after delivering the AS (Figure 1F, paired *t*-test, compared with baseline, 0–10 min: 1.475 ± 0.09572, *P* = 0.0025; 30–40 min: 1.026 ± 0.0616, *P* = 0.6891; 50–60 min: 1.015 ± 0.08825, *P* = 0.8691; *n* = 7 cells). The extent of LTP reversal was 93.95 ± 16.86% (Figure 1I, *n* = 7 cells).

Poo’s group reported that random flashes were effective in reversing LTP only when they were presented within the first 20 min after the induction of LTP in tectal neurons of Xenopus (Zhou et al., 2003). Therefore, we examined the time window of the LTP reversal during which the AS was delivered after 30 min of LTP induction. We found that the extent of LTP reversal was 7.211 ± 28.15% after 30 min of LTP induction (Figures 1G,I; unpaired *t*-test, compared with the extent of LTP reversal when AS was delivered after 10 min of LTP induction, *P* = 0.0185, *n* = 5 cells). AS could obviously reverse LTP within 10 min after the induction of LTP in adult mature DGCs. Taken together, these results showed that LTP could be effectively reversed in a time-dependent pattern.

We sought to reverse LTP with the single stimuli model of presynaptic stimulation or postsynaptic spikes to test whether presynaptic and postsynaptic stimuli are both required for inducing LTP reversal. The presynaptic stimulation could transiently reverse LTP within 10 min of LTP induction, but it slowly recovered to the original LTP level after 20 min (Figure 1J, solid circles, *n* = 6 cells). Postsynaptic spikes made no difference in LTP during the first 10-min period after the induction of LTP (open circle, *n* = 6 cells). Therefore, both presynaptic and postsynaptic mechanisms are involved in LTP reversal in the DG. Usually, LTP reversal can be induced by low-frequency stimulation (LFS) both *in vitro* and *in vivo* (HzFroc et al., 2000; Sugita et al., 2016), in which LFS is ineffective at inducing LTD (Huang et al., 1999). This phenomenon of LTP reversal might be the distinct nature of LTD. Therefore, we first tested the effect of AS on basal synaptic plasticity. We recorded the EPSPs before and after giving the AS to test the influence on the basal synaptic transmission. As we could see, AS failed to induce stabilized LTD, although the EPSP amplitude decreased during the first 10-min period (open circles, *n* = 8 cells). We found that 1 Hz LFS (900 pulses) failed to induce LTP reversal (open circle, *n* = 5 cells), but it could induce stable LTD (Figure 1K, solid circles, *n* = 7 cells).

### LTP and LTP Reversal Varies With Developmental Stage of the Adult-Born DGCs in the Hippocampus

**FIGURE 2:**
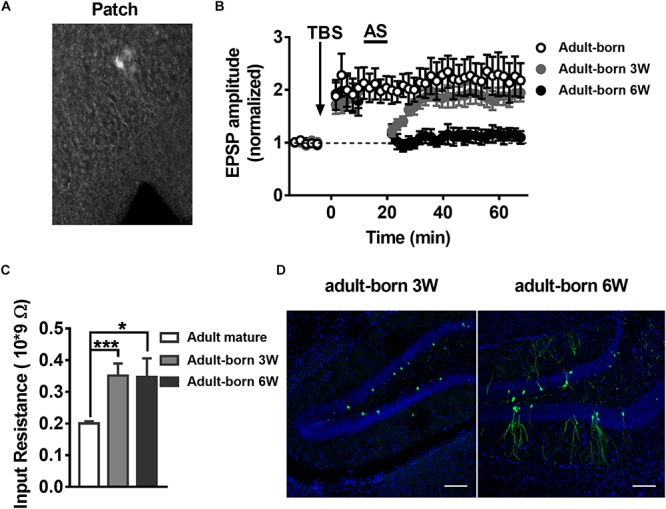
LTP and LTP reversal vary with the developmental stage of the adult-born DGCs in the hippocampus. **(A)** A photograph of the EPSP recording setup by patch-clamp in adult-born DGCs expressing GFP from retrovirus injected mice. **(B)** LTP in adult-born DGCs (open circles) and LTP reversal in adult-born 3-week-old (gray-solid circles) and adult-born 6-week-old (black-solid circles) DGCs. LTP was successfully reversed in adult-born 6-week-old DGCs but not in adult-born 3-week-old DGCs after delivering the AS. **(C)** Input resistance in adult mature DGCs (white bar) and adult-born 3-week-old (gray bar) and adult-born 6-week-old (black bar) DGCs. Input resistance changed with the development of adult-born DGCs. Values represent the mean ± SEM (^∗^*p* < 0.05; ^∗∗∗^*p* < 0.001; unpaired *t*-test). **(D)** Confocal images of GFP^+^ flourscence (green) and DAPI staining (blue) in newborn 3-week-old and newborn 6-week-old DGCs from the adult hippocampus. Scale bar, 100 μm.

It has been reported that synaptic plasticity dynamically changes during the development of adult-born neurons, especially LTP (Ge et al., 2007), although the changes in LTP reversal during maturation of adult-born DGCs in the hippocampus remain unknown. To test LTP and LTP reversal at different stages during the development of adult newborn DGCs, we labeled newborn neurons in the adult hippocampus with stereotaxically injected retroviruses (RV) expressing green fluorescent protein (GFP) into the DG of adult mice (for details see Experimental Procedures). GFP^+^ newborn DGCs in fresh brain slices prepared from retrovirus-infected adult mice were recorded with whole-cell patch-clamp (Figure 2A). Obvious LTP of EPSPs was induced with TBS in GFP^+^ DGCs after 3 weeks of RV infection (Figure 2B, paired *t*-test, compared with baseline, 0–10 min: 2.022 ± 0.3148, *P* = 0.0228; 30–40 min: 2.196 ± 0.3054, *P* = 0.0112; gray-solid circles, *n* = 6 cells), when most newborn DGCs are already fully integrated into the existing circuitry (Esposito et al., 2005; Ge et al., 2006). We recorded EPSPs from GFP^+^ DGCs at 6 weeks after RV infection (Figure 2B, black-solid circles, *n* = 6 cells) when newborn neurons are nearly entirely morphologically (Zhao et al., 2006) and physiologically matured (Laplagne et al., 2006). Interestingly, LTP was reversed successfully in newborn 6-week-old DGCs after delivering the AS post-10 min of LTP induction (paired *t*-test, compared with baseline, 0–10 min: 1.895 ± 0.275, *P* = 0.0226; 30–40 min: 1.087 ± 0.121, *P* = 0.5036; *n* = 6 cells), but newborn 3-week-old DGCs failed to reverse the LTP when the same AS paradigm was transmitted (paired *t*-test, compared with baseline, 0–10 min: 1.817 ± 0.1083, *P* = 0.0006; 30–40 min: 1.87 ± 0.1638, *P* = 0.0032; *n* = 6 cells). These results demonstrated that LTP reversal gradually emerged during the maturation of adult-born DGCs.

It has been reported that adult-born DGCs present different electrophysiological properties, including higher input resistance and lower thresholds for the induction of LTP than mature DGCs (Mu et al., 2011; Marin-Burgin et al., 2012; Dieni et al., 2016). Thus, we quantified the input resistance of DGCs. Consistent with previous reports, the mean input resistance of adult-born DGCs was significantly larger than adult mature DGCs (Figure 2C, unpaired *t*-test, compared with adult mature DGCs, adult mature DGCs: 0.2003 ± 0.006771 GΩ, *n* = 8 cells; adult-born 3 W: 0.3514 ± 0.03797 GΩ, *P* = 0.0007, *n* = 6 cells; adult-born 6 W: 0.3469 ± 0.05898 GΩ, *P* = 0.0140, *n* = 6 cells). A previous study reported that newborn 2–3-week-old DGCs begin to receive excitatory input from entorhinal cortical perforant pathway synapses, and their physiology and anatomy begin to approach maturation at 4–8 weeks, coupled with slowly developing dendritic arborizations and axonal projections in adult brain (Aimone et al., 2014). We first observed morphological changes during newborn DGC maturation in the adult hippocampus. Here, we can see that newborn 3-week-old DGCs extend their apical dendrites into the molecular layer, but newborn 6-week-old neurons displayed abundant dendrite arborizations in the molecular layer in the DG (Figure 2D).

### Developmental Alterations in Adult Mouse 3-Week-Old and 6-Week-Old Newborn DGC NMDAR Subunit Composition

**FIGURE 3:**
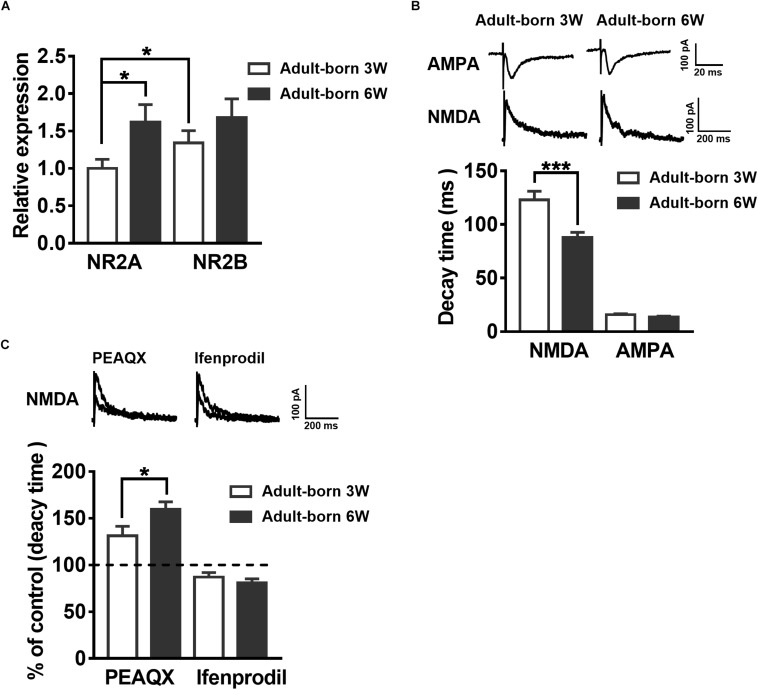
Developmental alterations in NMDAR subunit composition in adult mouse newborn DGCs. **(A)** NR2AR and NR2BR mRNA relative expression in newborn 3-week-old and 6-week-old DGCs of adult mice. Values represent the mean ± SEM (^∗^*p* < 0.05; *t*-test). **(B)** The AMPA- and NMDA-mediated EPSCs recorded under whole-cell voltage-clamp (Vm = –70 mV for AMPA-EPSCs, Vm = + 40 mV for NMDA-EPSCs) from adult-born 3-week-old and adult-born 6-week-old DGCs. CNQX (10 μM) and PTX (50 μM) were delivered in the perfused ACSF solution throughout NMDA-EPSC recordings. Shown in the upper row are representative example traces of AMPA- and NMDA-EPSCs recorded under the whole-cell voltage-clamp. The lower row shows the statistical analysis of the AMPA- and NMDA-EPSC decay time in adult-born 3-week-old (white bar) and adult-born 6-week-old (black bar) DGCs. Bottom row, the summary data demonstrate that the decay time of NMDA-EPSCs becomes slower in adult-born 3-week-old than in adult-born 6-week-old DGCs, whereas the decay time of AMPA-EPSCs does not obviously change during the development of adult newborn DGCs. Values represent the mean ± SEM (^∗∗∗^*p* < 0.001; unpaired *t*-test). **(C)** Shown in the top row are typical sample traces of NMDA-EPSCs recorded under whole-cell voltage-clamp before and after perfusion with PEAQX or ifenprodil in adult mouse newborn 3-week-old DGCs. Bottom row, quantification of the decay time of NMDA-EPSCs in newborn 3-week-old and newborn 6-week-old DGCs after PEAQX and ifenprodil administration. Values represent the mean ± SEM (^∗^*p* < 0.05; unpaired *t*-test).

NMDARs are important for adult newborn DGC maturation in the hippocampus (Ge et al., 2007; Mu et al., 2015). Acute treatment with NMDA rapidly decreases the number of newborn neurons, whereas NMDAR antagonist injection increases the birth of DGCs in the rat hippocampus (Cameron et al., 1995). We sought to determine the NMDAR subunit distribution and contribution alterations during newborn DGC development in the adult hippocampus. It was reported that NR2A mRNA expression increases in association with downregulation of NR2B mRNA expression during neuron maturation in cultured cerebellar granule cells (Vallano et al., 1996). We detected NR2AR and NR2BR mRNA in adult mouse 3-week-old and 6-week-old newborn DGCs by single-cell real-time PCR. The NR2AR mRNA expression level of newborn 3-week-old DGCs was standardized as a reference. The NR2AR mRNA relative expression of newborn 6-week-old DGCs was distinctly higher than that of newborn 3-week-old DGCs (Figure 3A, unpaired *t*-test, *P* = 0.0303, adult-born 3 W: 1 ± 0.121, *n* = 16 mice; adult-born 6 W: 1.619 ± 0.2337, *n* = 18 mice) in adult mice. There was no difference in NR2BR mRNA relative expression between the two groups of adult-born DGCs in the hippocampus (unpaired *t*-test, *P* = 0.2699, 3-week-old adult-born: 1.341 ± 0.1634, *n* = 16 mice; 6-week-old adult-born: 1.682 ± 0.2469, *n* = 18 mice).

It has been reported that the NMDA-EPSC decay time decreases with increased expression of the NR2A subunit during development (Roberts and Ramoa, 1999). The influence of the NMDAR subunit composition can shift the kinetics of NMDAR-mediated EPSCs (Vicini et al., 1998). Therefore, we recorded AMPA-EPSCs and NMDA-EPSCs in newborn 3-week-old and newborn 6-week-old DGCs to test the NMDAR subunits constitute. AMPAR-mediated EPSCs were recorded at −70 mV in the presence of PTX (50 μM) in ACSF. NMDAR-mediated EPSCs were pharmacologically isolated by CNQX (10 μM) in +40 mV (Mu et al., 2015). The NMDAR-mediated EPSC decay time in newborn 6-week-old DGCs was significantly smaller than that in newborn 3-week-old DGCs (Figure 3B, unpaired *t*-test, *P* = 0.0005, adult-born 3 W: 123.1 ± 7.973 ms, *n* = 17 cells; adult-born 6 W: 87.81 ± 4.79 ms, *n* = 18 cells). There was no significant difference in AMPA-EPSC decay times between the two groups (unpaired *t*-test, *P* = 0.0826, adult-born 3-week-old: 15.78 ± 0.8935 ms, *n* = 17 cells; adult-born 6-week-old: 13.59 ± 0.8354 ms, *n* = 18 cells). In addition, NMDA-EPSC decay times significantly increased after perfusion with the NR2AR blocker PEAQX (0.4 μM) in newborn 3-week-old adult hippocampus DGCs compared with the decay times before perfusion (Figure 3C, *P* < 0.0001, paired *t*-test, 131.3 ± 10.08% of decay times before perfusion, *n* = 7 cells). In contrast, perfusing the NR2BR blocker ifenprodil (3 μM) in adult-born 3-week-old DGCs (paired *t* test, *P* < 0.0001, 87.07 ± 4.804% of decay times before perfusion, *n* = 10 cells). Similarly, NMDA-EPSC decay times significantly increased after perfusion with the NR2AR blocker PEAQX in adult-born 6-week-old DGCs (paired *t* test, *P* < 0.0001, 159.6 ± 8.033% of decay times before perfusion, *n* = 7 cells) compared with before perfusion. In contrast, perfusion of the NR2BR blocker ifenprodil (paired *t*-test, 81.01 ± 4.164% of decay times before perfusion, *P* < 0.0001, *n* = 8 cells) in newborn 6-week-old DGCs. Nevertheless, the percentage change in NMDA-EPSC decay times after administration of PEAQX was significantly higher in newborn 6-week-old DGCs than in newborn 3-week-old DGCs (unpaired *t*-test, *P* = 0.0487), which indicated a higher proportion of NR2AR in NMDAR subunits of newborn 6-week-old DGCs than in those of newborn 3-week-old DGCs in the adult brain.

### LTP Reversal and the Expression of NMDAR Subunits in the DG Change With Postnatal Individual Development

**FIGURE 4:**
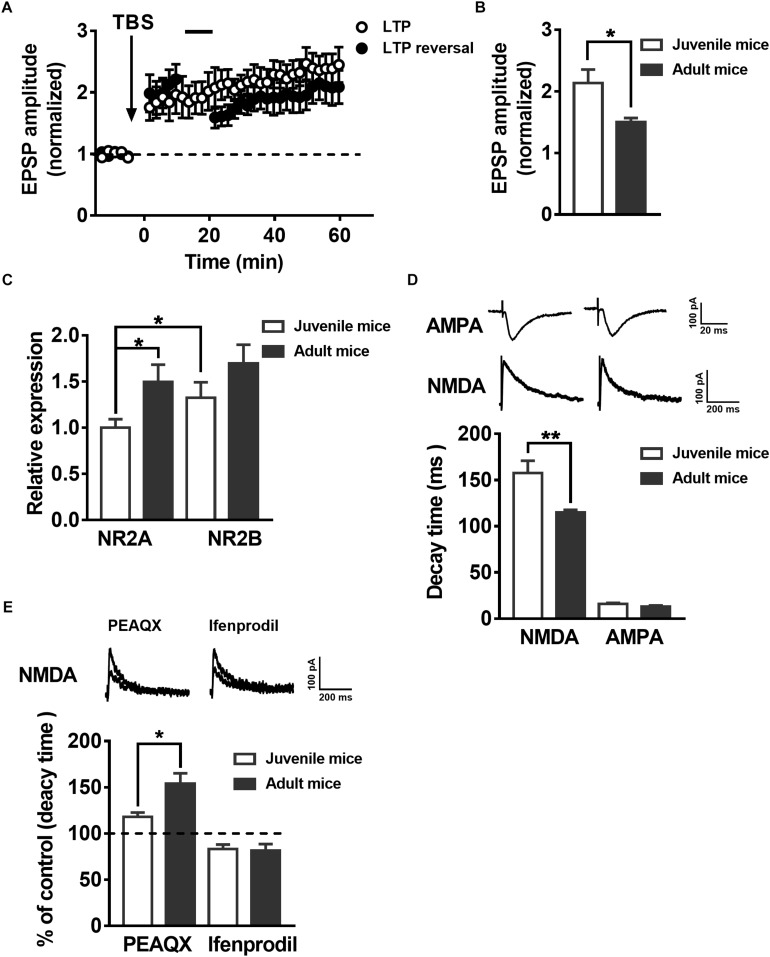
LTP reversal and the expression of NMDAR subunits in the DG change with postnatal individual development. **(A)** LTP (open circles, *n* = 7 cells) and LTP reversal (solid circles, *n* = 7 cells) in juvenile mouse DGCs. **(B)** Summary of the mean LTP amplitude during LTP recording (0–60 min) in juvenile mouse mature DGCs and adult mouse mature DGCs. Values represent the mean ± SEM (^∗^*p* < 0.05; unpaired *t*-test). **(C)** mRNA expression of the NMDAR subunits NR2AR and NR2BR in the DG of juvenile and adult mice. The relative expression of DG NR2A mRNA in adult mice was significantly higher than in juvenile mice. Values represent the mean ± SEM (^∗^*p* < 0.05; *t*-test). **(D)** Shown in the top row are typical example traces of AMPA- and NMDA-EPSCs recorded under whole-cell voltage-clamp. Bottom row, the statistical analysis demonstrates that the decay time of NMDA-EPSCs is slower in juvenile (white bar) than in adult (black bar) mouse mature DGCs, whereas the decay time of AMPA-EPSCs did not have an obvious change during individual development. Values represent the mean ± SEM (^∗∗^*p* < 0.01; unpaired *t*-test). **(E)** Shown in the top row are typical sample traces of NMDA-EPSCs before and after perfusing the PEAQX or ifenprodil in adult mouse mature DGCs. The bottom row shows the quantification of decay time of NMDA-EPSCs in juvenile (white bar) and adult (black bar) mouse mature DGCs after PEAQX and ifenprodil perfusion. Values represent the mean ± SEM (^∗^*p* < 0.05; unpaired *t*-test).

To test whether synaptic plasticity related to LTP and LTP reversal during the development of postnatal mice presents similar changes with the maturation of adult-born neurons in the hippocampus, we recorded the EPSPs in juvenile mouse (postnatal 19–21 days) mature DGCs and adult mouse (postnatal 60–70 days) mature DGCs. A previous study suggested the reversal of LTP changes with development in postnatal individuals (Kramár and Lynch, 2003). Here, we showed that the same AS protocol that reversed the LTP in adult mice mature neurons could not reverse the LTP in juvenile mice mature DGCs under the same conditions (Figure 4A, paired *t*-test, compared with the baseline, 0–10 min: 2.047 ± 0.2534, *P* = 0.0061; 30–40 min: 1.887 ± 0.2063, *P* = 0.0051; black-solid circles, *n* = 7 cells). The normalized EPSP amplitude of juvenile mouse mature DGCs was maintained at a stable level after delivering the TBS (Figure 4A, paired *t*-test, comparing the normalized EPSP amplitude at 0–10 min with 30–40 min, *P* = 0.1440, 0–10 min: 1.864 ± 0.2663; 30–40 min: 2.197 ± 0.2209; open circle, *n* = 7 cells). We also found the mean LTP amplitude of juvenile mouse mature DGCs during the whole LTP (0–60 min) was significantly larger than that of adult mouse mature DGCs [Figure 4B, unpaired *t*-test, *P* = 0.0269, juvenile mice: 2.133 ± 0.2207, *n* = 7 cells (Figure 4A, open circle); adult mice: 1.503 ± 0.06319, *n* = 6 cells (Figure 1D, open circle)]. NMDARs are important for synaptic plasticity during postnatal individual development (Sheng et al., 1994). It has been reported that the NMDAR subunits NR2AR and NR2BR change with postnatal individual development of the brain (Wenzel et al., 1997; Liu X.B. et al., 2004). To explore whether the NMDAR subunits NR2A and NR2B changed during postnatal individual development, we detected the NR2A and NR2B mRNA expression levels in hippocampal DG tissue using real-time PCR. We found that the relative expression of NR2A in the adult DG was significantly higher than that in the juvenile DG (Figure 4C, unpaired *t* test, *P* = 0.0225, juvenile mice: 1 ± 0.09187, *n* = 11 mice; adult mice: 1.494 ± 0.1888, *n* = 9 mice). The NR2BR mRNA relative expression level showed no difference between the juvenile DG and the adult DG (unpaired *t*-test, *P* = 0.1715, juvenile mice: 1.326 ± 0.1672, *n* = 11 mice; adult mice: 1.696 ± 0.2026, *n* = 9 mice). The reliability of the real-time PCR was confirmed by running a gel imaging analysis (Supplementary Figure S2). In addition, evoked EPSCs were recorded with whole-cell voltage-clamp from both juvenile and adult hippocampus DGCs. As reported previously, NMDA-EPSCs from adult Sprague Dawley rat hippocampi exhibit much faster decay times than juvenile rats (Rumbaugh and Vicini, 1999). Here, we observed that NMDA-EPSC decay kinetics are significantly faster in adult hippocampus DGCs than in juvenile hippocampus DGCs (Figure 4D, unpaired *t*-test, *P* = 0.0066, juvenile mice: 157.5 ± 13.37 ms, *n* = 9 cells; adult mice: 114.9 ± 2.895 ms, *n* = 9 cells), which further demonstrated that NR2AR subunit expression was apparently higher in juvenile DG NMDARs than in adult DG NMDARs. There were no obvious differences in AMPA-EPSC decay times between juvenile and adult DGCs (unpaired *t*-test, *P* = 0.1189, juvenile mice: 15.94 ± 1.212 ms, *n* = 9 cells; adult mice *t*: 12.87 ± 1.417 ms, *n* = 9 cells). Similar to the changes during maturation of adult newborn DGCs in the hippocampus, the percentage increase in NMDA-EPSC decay time in the adult mouse mature DGCs was significantly higher than that in juvenile mouse mature DGCs after perfusion with PEAQX (Figure 4E, unpaired *t*-test, *P* = 0.0190, adult mice: 154 ± 11.34% of the decay time before perfusion, *n* = 7 cells; juvenile mice: 117.9 ± 4.921% of the decay time before perfusion, *n* = 6 cells). In contrast, the decreased NMDA-EPSC decay time percent in the adult mouse mature DGCs was not significantly different between juvenile mouse mature DGCs after perfusion with ifenprodil (unpaired *t*-test, *P* = 0.8407, adult mice: 81.58 ± 7.064% of the decay time before perfusion, *n* = 7 cells; juvenile mice: 83.28 ± 4.748% of the decay time before perfusion, *n* = 8 cells). These results indicated that the proportion of NR2AR in NMDAR subunits was greater in juvenile mouse mature DGCs than in juvenile mouse mature DGCs in the adult brain.

### Differential Modulation of LTP Reversal in the DGCs by NMDAR Subtypes

**FIGURE 5:**
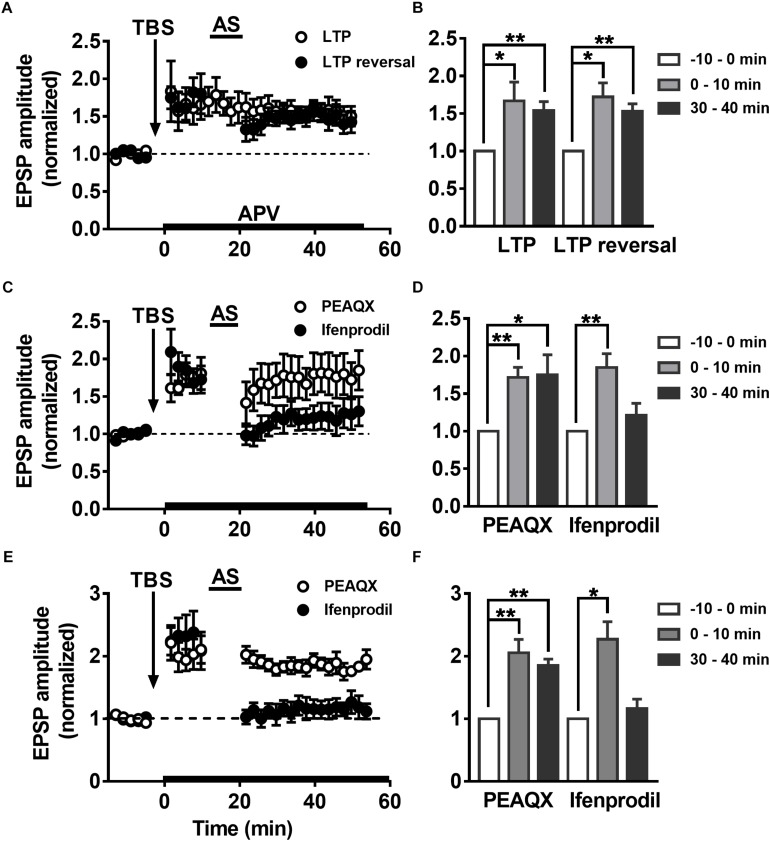
Differential modulation of LTP reversal in the DGCs by NMDAR subtypes. **(A)** LTP (open circles) and LTP reversal (solid circles) recorded from adult mature DGCs in the presence of NMDAR blocker APV. APV was administered at 10 min post-LTP induction, indicated by the black horizontal bar. **(B)** Summary of the means at different time points during recordings of LTP and LTP reversal after perfusing APV in **(A)**. APV had no obvious effect in LTP maintaining, but it blocked the reversal of LTP induced by TBS. Values represent the mean ± SEM (^∗^*p* < 0.05, ^∗∗^*p* < 0.01; paired *t*-test). **(C)** LTP reversal recorded from adult mature DGCs in the presence of NR2AR antagonist PEAQX (0.4 μM, open circles) or NR2BR antagonist ifenprodil (3 μM, solid circles). The antagonists were delivered after the induction of LTP in adult mature DGCs, indicated by the black horizontal bar. **(D)** Summary of the means at the different time points during recording of the LTP and the LTP reversal after perfusing with PEAQX or ifenprodil in **(C)**. LTP reversal obviously inhibited by PEAQX but not ifenprodil. Values represent the mean ± SEM (^∗^*p* < 0.05, ^∗∗^*p* < 0.01; paired *t*-test). **(E)** LTP reversal recorded from adult-born 6-week-old DGCs in the presence of PEAQX (open circles) or ifenprodil (solid circles). **(F)** Summary of the means at the different time points in **(E)**. Bath perfusion with PEAQX, but not ifenprodil inhibited the LTP reversal induced by AS. Values represent the mean ± SEM (^∗^*p* < 0.05, ^∗∗^*p* < 0.01; paired *t*-test).

Some groups have reported that LTP reversal is significantly blocked by NMDAR antagonists (Fujii et al., 1991; Wagner and Alger, 1995). It was reported that selectively blocking NMDAR subunit NR2B abolishes the induction of LTD but not LTP, but inhibition of NR2A results in the opposite phenomenon (Liu L. et al., 2004). The role of the NMDAR subunits NR2A and NR2B in LTP reversal is not clear. To explore how the NMDAR subunits NR2AR and NR2BR mediate changes in LTP reversal in hippocampal DGCs, the NMDAR-specific antagonist APV (50 μM) was perfused during the first 10 min after LTP was successfully induced in adult mature DGCs. As reported previously, APV had no effect on LTP maintenance after 10 min of LTP induction (Figures 5A,B, paired *t*-test, comparing the normalized EPSP amplitude at 0–10 min with 30–40 min, *P* = 0.5013, 0–10 min: 1.667 ± 0.2516, *n* = 8 cells; 30–40 min: 1.54 ± 0.1195, *n* = 8 cells). However, the application of APV while inducing LTP reversal by delivering the AS obviously blocked the induction of LTP reversal (paired *t*-test, compared with baseline, 0–10 min: 1.721 ± 0.1878, *P* = 0.0121; 30–40 min: 1.533 ± 0.09779, *P* = 0.0028; *n* = 6 cells). In addition, the NR2A blocker PEAQX also obviously inhibited LTP reversal in adult mature DGCs (Figures 5C,D, paired *t*-test, compared with baseline, 0–10 min: 1.716 ± 0.1338, *P* = 0.0031; 30–40 min: 1.752 ± 0.2655, *P* = 0.0366; *n* = 6 cells), but the NR2B blocker ifenprodil did not (paired *t*-test, compare with the baseline, 0–10 min: 1.85 ± 0.182, *P* = 0.0095; 30–40 min: 1.215 ± 0.1571, *P* = 0.2436; *n* = 5 cells). PEAQX, but not ifenprodil, significantly blocked LTP reversal in adult-born 6-week-old DGCs (Figures 5E,F, paired *t*-test, normalized EPSP amplitude at 30–40 min compared with baseline, PEAQX: 1.853 ± 0.1001, *P* = 0.0010, *n* = 5 cells; ifenprodil: 1.162 ± 0.151, *P* = 0.3450; *n* = 5 cells), which is in accordance with the result in adult mature DGCs. In conclusion, the NMDAR subunit NR2AR mediates LTP reversal in the development of DGCs.

## Discussion

Long-term potentiation reversal leads to prior acquired memory deficiency (Kim and Cho, 2017), which is likely to be the mechanism by which postlearning interfering stimuli induce forgetting (Wixted, 2004). Our finding demonstrated that the susceptibility to LTP reversal progressively increases during the development of postnatal individuals and maturation of adult-born neurons, which was coupled with NMDA receptor subunit NR2A expression augmentation. In addition, the reason for the increase in LTP reversal susceptibility is the increase in NR2A relative expression. LTP reversal can be achieved within 10 min of induction (Huang et al., 1999; Staubli and Scafidi, 1999). Figure 1 shows the induction of LTP and LTP reversal in the DG of the adult mouse hippocampus by TBS and AS, which is distinct from a protein synthesis-dependent LTP consolidation that requires several hours to begin (Nguyen et al., 1994). However, it has been reported that new dendritic structures emerge approximately 15–30 min after LTP induction in the hippocampus CA1 (Engert and Bonhoeffer, 1999; Maletic-Savatic et al., 1999). It is believed that LFS (2 Hz) or theta-frequency stimulation (5 Hz) can effectively reverse LTP in the brain (Zhou and Poo, 2004). LTP reversal occurred at the input that received the LTP reversal stimuli but not at another potentiated input to the same postsynaptic cell (Muller et al., 1995). Our experiment adopted a modified AS stimuli pattern to simulate the spontaneous activity of tectal neurons during LTP reversal in the visual system (Zhou et al., 2003) to investigate the physiological significance of LTP reversal, which effectively reversed the LTP in the adult hippocampus DG region (Figure 1). In addition, this AS pattern revealed that both pre- and postsynaptic mechanisms are needed to induce LTP reversal. The same AS paradigm for reversing LTP could not induce LTD in adult mature DGCs (Figure 1K). However, LTD could be induced by LFS (1 Hz) in the presence of PTX, which could not reverse LTP in the adult brain (Figure 1L). These findings further illuminated that LTP reversal is a nervous system phenomenon involving persistent changes in synaptic responsiveness, which differs from LTD (Wagner and Alger, 1996).

Long-term potentiation amplitude and the extent of LTP reversal change during development of postnatal individual and maturation of newborn neurons (Wagner and Alger, 1995; Kramár and Lynch, 2003; Ge et al., 2007). The dynamic changes in the mRNA expression of NMDAR subunits NR2A and NR2B during the maturation of adult-born DGCs, in which LTP reversal presented dramatic corresponding changes, have not been previously reported. We used single-cell real-time PCR to test the NMDAR subunit NR2A mRNA expression and whole-cell patch-clamp to recording the NMDA-mediated EPSCs in adult-born 3-week-old and adult-born 6-week-old DGCs. We can see that NR2A subunit expression increased and the decay time decreased in adult-born 6-week-old DGCs (Figure 3), which was coupled with successfully induced LTP reversal compared with the failure to induce LTP reversal in adult-born 3-week-old DGCs (Figure 2). Similar to changes in the maturation of adult-born DGCs, synaptic plasticity presented relevant changes during development of postnatal individuals (Kramár and Lynch, 2003). However, the differences in stimulation protocols and brain regions may account for the discrepancy between the experiments regarding the finding that LTP reversal susceptibility increased with increasing age (Figure 4B). During the development of postnatal individuals and the maturation of adult-born neurons, NR2A increased coupled with LTP reversal under the same stimulus pattern (Figures 2–4). Whole-cell patch-clamp to record the NMDA-mediated EPSCs showed that the decay time of NMDA-EPSCs decreased with the augmentation of NMDAR subunit NR2A mRNA expression during individual postnatal development (Figure 4). We found that NR2A subunit expression was increased and activity-dependent NMDAR responses were decreased, as previously reported in the visual cortex during individual postnatal development (Carmignoto and Vicini, 1992; Liu X.B. et al., 2004).

NR2A-containing NMDARs produce faster (Monyer et al., 1994; Flint et al., 1997) and smaller EPSCs (Barria and Malinow, 2002), which may influence the amount of Ca^2+^ entering the intracellular space. It has been hypothesized that these properties could be responsible for the reduced synaptic plasticity observed in the brain during individual postnatal development (Carmignoto and Vicini, 1992; Crair and Malenka, 1995) or after learning (Quinlan et al., 2004). It is known that the patterns of intracellular Ca^2+^ rise dictate the direction of NMDAR-dependent synaptic plasticity (Yang et al., 1999). Higher levels of NMDAR activation produce LTP, while low levels of activation induce LTD (Artola and Singer, 1993). The reversal of LTP immediately after LTP induction depends on a small amount of Ca^2+^ entering the intracellular space through NMDARs during stimuli, which in turn activates protein phosphatase 1 (PP-1) to change the cytoskeleton and gene transcription to mediate synaptic plasticity (Huang and Hsu, 2001; Zhou and Poo, 2004). The amount of Ca^2+^ entering the intracellular space through NMDARs may be decided by a subunit substitution in the NMDARs, which influences the decay time of NMDARs. A previous study demonstrated that the decay time is significantly slower for NR1/NR2B NMDARs than for NR1/NR2A NMDARs (Vicini et al., 1998), in which the shorter NMDAR decay time may decrease the amount of Ca^2+^ entering the intracellular space. NMDAR activation has been shown to play a role in learning and memory (Paoletti et al., 2013) as well as in adult neurogenesis (Deisseroth et al., 2004; Mu et al., 2015). Adult neurogenesis and NMDARs mediate bidirectional hippocampal synaptic plasticity (Liu L. et al., 2004; Massa et al., 2011). LTP reversal can be mimicked by a brief application of NMDA during the initial susceptible phase of LTP in the CA1 region of hippocampus slices (Lee et al., 1998). It has been shown that NMDARs mediate LTP reversal (Kramár and Lynch, 2003; Zhou et al., 2003), similar to our findings, and NMDAR-specific antagonist APV significantly inhibited the reversal of LTP in DGCs (Figures 5A,B). NR2A- and NR2B-containing NMDARs are the major subtypes of NMDARs expressed in the hippocampus (Monyer et al., 1994), each playing distinct roles in different forms of synaptic plasticity; NR2A-containing NMDARs are required for LTP, whereas NR2B-containing NMDARs are required for LTD in rat brains (Liu L. et al., 2004; Massey et al., 2004). We found that the NMDAR subunit NR2A antagonist PEAQX obviously depressed the reversal of LTP in adult hippocampal mature DGCs and in adult-born 6-week-old DGCs (Figures 5C–F).

Beside NMDA receptors, mechanisms underlying LTP reversal are presumably related to the reversal of cellular changes associated with LTP induction. AMPA receptors are phosphorylated at Ser831 on GluR1 subunit by CaMKII. Such phosphorylation is responsible for the increase in the conductance of AMPA receptors after LTP (Benke et al., 1998). The LTP-induced phosphorylation of Ser831 is reversed during LTP reversal (Lee et al., 2000; Huang et al., 2001). Incubation of the tectal neuron with a selective PP-1/2A blocker okadaic acid eliminated the reversal of LTP in a developing visual system (Zhou et al., 2003). The similar phenomena are shown in the hippocampus (O’Dell and Kandel, 1994; Huang et al., 1999; Kang-Park et al., 2003). Taken together, the interaction between kinases and phosphatases can determine whether LTP is induced and how long it will last. If the phosphatase activity overwhelms that of kinases, LTP can be reversed. The possibility remains that additional mechanisms may also contribute to LTP reversal. Application of metabotropic glutamate (mGlu) receptors with mGlu5 subtype–selective antagonists prevented the LTP reversal in the hippocampus (Qi et al., 2013). Increasing extracellular adenosine may underlie the LTP reversal by inhibiting the cAMP-dependent signaling cascade induced LTP via the A1 receptor subtype coupled to Gi/o proteins to prevent the PKA signaling (Huang et al., 1999).

A previous study reported that reversal of LTP by spontaneous activity may serve as a protective mechanism against persistent synaptic changes triggered by an incidental insignificant event in the developing visual system (Zhou et al., 2003). Therefore, we speculated that LTP reversal may function as an eraser of LTP to modify synaptic plasticity, which may mediate forgetting. NR2A-mediated reversal of LTP was impaired in the hippocampal adult-born DGCs, which might cause adult-born DGCs not eliminate the subsequent transient memory; namely, adult-born DGCs could resist the incidental episodes of uncorrelated activity to form stabilized LTP. Similar to LTP reversal deficiency in juvenile mice, juvenile animals learn faster than adult animals. A recent report showed by using hippocampus-dependent object-location memory and contextual fear conditioning tasks confirmed that juvenile mice displayed deficits in long-term memory retention compared with adult mice (Tsai et al., 2018), which was probably because juvenile mice have insufficient memory encoding (Ge et al., 2019).

Synaptic plasticity dynamically changes during adult neurogenesis (Ge et al., 2007). Adult-born DGCs mediate pattern separation, whereas mature DGCs facilitate pattern completion (Clelland et al., 2009; Nakashiba et al., 2012). Our work indicates that LTP reversal is impaired in hippocampal adult-born 3-week-old DGCs, which may make it easier for newborn DGCs to build reliable connections between adult-born cells to participate in memory consolidation (Kitamura et al., 2009). It was reported that LTP reversal disappeared in a schizophrenia mouse model (Shamir et al., 2012). Schizophrenia may be caused by subtle differences in neural development leading to erroneous neuronal connections (Lewis and Levitt, 2002). In addition, a recent report claimed that hippocampal adult-born DGCs were reduced and working memory was impaired in a schizophrenia model (Ouchi et al., 2013). We wondered whether deficiency of LTP reversal in adult-born DGCs of adult mice contributes to cognitive and behavioral impairments in schizophrenia. It remains unknown whether impaired adult hippocampal neurogenesis is a causal factor underlying relevant pathology, including schizophrenia. Nevertheless, targeting adult-born neurons could be a potential therapeutic strategy for schizophrenia (Christian et al., 2014). Additional studies are needed to elucidate the molecular mechanisms of LTP reversal in adult-born DGCs and their relationship with forgetting and schizophrenia.

## Data Availability Statement

The raw data supporting the conclusions of this manuscript will be made available by the authors, without undue reservation, to any qualified researcher.

## Ethics Statement

The animal study was reviewed and approved by the Committee of Animal Care of Huazhong University of Science and Technology.

## Author Contributions

YM and NS conceived the project and designed the study. XT performed the experiments and wrote the first draft of the manuscript. NS was responsible for the validation of data. NS and YM were responsible for editing and reviewing the manuscript.

## Conflict of Interest

The authors declare that the research was conducted in the absence of any commercial or financial relationships that could be construed as a potential conflict of interest.

## Abbreviations

AS: associated stimulation.

## References

[B1] Aimone J. B. Li Y. Lee S. W. Clemenson G. D. Deng W. Gage F. H. (2014). Regulation and function of adult neurogenesis: from genes to cognition. *Physiol. Rev.* 94 991–1026. 10.1152/physrev.00004.2014 25287858 PMC4280160

[B2] Artola A. Singer W. (1993). Long-term depression of excitatory synaptic transmission and its relationship to long-term potentiation. *Trends Neurosci.* 16 480–487. 10.1016/0166-2236(93)90081-V 7507622

[B3] Barria A. Malinow R. (2002). Subunit-specific NMDA receptor trafficking to synapses. *Neuron* 35 345–353. 10.1016/s0896-6273(02)00776-6 12160751

[B4] Benke T. A. Lüthi A. Isaac J. T. Collingridge G. L. (1998). Modulation of AMPA receptor unitary conductance by synaptic activity. *Nature* 393 793–797. 10.1038/31709 9655394

[B5] Bliss T. V. Lomo T. (1973). Long-lasting potentiation of synaptic transmission in the dentate area of the anaesthetized rabbit following stimulation of the perforant path. *J. Physiol.* 232 331–356. 10.1113/jphysiol.1973.sp010273 4727084 PMC1350458

[B6] Boldrini M. Fulmore C. A. Tartt A. N. Simeon L. R. Pavlova I. Poposka V. (2018). Human hippocampal neurogenesis persists throughout Aging. *Cell Stem Cell* 22 e585. 10.1016/j.stem.2018.03.015 29625071 PMC5957089

[B7] Cameron H. A. McEwen B. S. Gould E. (1995). Regulation of adult neurogenesis by excitatory input and NMDA receptor activation in the dentate gyrus. *J. Neurosci.* 15 4687–4692. 10.1523/jneurosci.15-06-04687.1995 7790933 PMC6577705

[B8] Carmignoto G. Vicini S. (1992). Activity-dependent decrease in NMDA receptor responses during development of the visual cortex. *Science* 258 1007–1011. 10.1126/science.1279803 1279803

[B9] Christian K. M. Song H. Ming G. L. (2014). Functions and dysfunctions of adult hippocampal neurogenesis. *Annu. Rev. Neurosci.* 37 243–262. 10.1146/annurev-neuro-0710130-14134 24905596 PMC5531058

[B10] Clelland C. D. Choi M. Romberg C. Clemenson G. D. Jr. Fragniere A. Tyers P. (2009). A functional role for adult hippocampal neurogenesis in spatial pattern separation. *Science* 325 210–213. 10.1126/science.1173215 19590004 PMC2997634

[B11] Collingridge G. L. Peineau S. Howland J. G. Wang Y. T. (2010). Long-term depression in the CNS. *Nat. Rev. Neurosci.* 11 459–473. 10.1038/nrn2867 20559335

[B12] Crair M. C. Malenka R. C. (1995). A critical period for long-term potentiation at thalamocortical synapses. *Nature* 375 325–328. 10.1038/375325a0 7753197

[B13] Deisseroth K. Singla S. Toda H. Monje M. Palmer T. D. Malenka R. C. (2004). Excitation-neurogenesis coupling in adult neural stem/progenitor cells. *Neuron* 42 535–552. 10.1016/s0896-6273(04)00266-1 15157417

[B14] Dieni C. V. Panichi R. Aimone J. B. Kuo C. T. Wadiche J. I. Overstreet-Wadiche L. (2016). Low excitatory innervation balances high intrinsic excitability of immature dentate neurons. *Nat. Commun.* 7:11313. 10.1038/ncomms11313 27095423 PMC4843000

[B15] Dingledine R. Borges K. Bowie D. Traynelis S. F. (1999). The glutamate receptor ion channels. *Pharmacol. Rev.* 51 7–61. 10049997

[B16] Engert F. Bonhoeffer T. (1999). Dendritic spine changes associated with hippocampal long-term synaptic plasticity. *Nature* 399 66–70. 10.1038/19978 10331391

[B17] Esposito M. S. Piatti V. C. Laplagne D. A. Morgenstern N. A. Ferrari C. C. Pitossi F. J. (2005). Neuronal differentiation in the adult hippocampus recapitulates embryonic development. *J. Neurosci.* 25 10074–10086. 10.1523/JNEUROSCI.3114-05.2005 16267214 PMC6725804

[B18] Flint A. C. Maisch U. S. Weishaupt J. H. Kriegstein A. R. Monyer H. (1997). NR2A subunit expression shortens NMDA receptor synaptic currents in developing neocortex. *J. Neurosci.* 17 2469–2476. 10.1523/jneurosci.17-07-02469.1997 9065507 PMC6573498

[B19] Fujii S. Saito K. Miyakawa H. Ito K. Kato H. (1991). Reversal of long-term potentiation (depotentiation) induced by tetanus stimulation of the input to CA1 neurons of guinea pig hippocampal slices. *Brain Res.* 555 112–122. 10.1016/0006-8993(91)90867-u 1681992

[B20] Ge M. Song H. Li H. Li R. Tao X. Zhan X. (2019). Memory susceptibility to retroactive interference is developmentally regulated by NMDA Receptors. *Cell Rep.* 26 2052–2063.e4. 10.1016/j.celrep.2019.01.098 30784588

[B21] Ge S. Goh E. L. Sailor K. A. Kitabatake Y. Ming G. L. Song H. (2006). GABA regulates synaptic integration of newly generated neurons in the adult brain. *Nature* 439 589–593. 10.1038/nature04404 16341203 PMC1420640

[B22] Ge S. Yang C. H. Hsu K. S. Ming G. L. Song H. (2007). A critical period for enhanced synaptic plasticity in newly generated neurons of the adult brain. *Neuron* 54 559–566. 10.1016/j.neuron.2007.05.002 17521569 PMC2040308

[B23] Gould E. McEwen B. S. Tanapat P. Galea L. A. Fuchs E. (1997). Neurogenesis in the dentate gyrus of the adult tree shrew is regulated by psychosocial stress and NMDA receptor activation. *J. Neurosci.* 17 2492–2498. 10.1523/jneurosci.17-07-02492.1997 9065509 PMC6573503

[B24] Hesse G. W. Teyler T. J. (1976). Reversible loss of hippocampal long term potentiation following electroconvulsive seizures. *Nature* 264 562–564. 10.1038/264562a0 1004596

[B25] Huang C. C. Hsu K. S. (2001). Progress in understanding the factors regulating reversibility of long-term potentiation. *Rev. Neurosci.* 12 51–68. 11236065 10.1515/revneuro.2001.12.1.51

[B26] Huang C. C. Liang Y. C. Hsu K. S. (1999). A role for extracellular adenosine in time-dependent reversal of long-term potentiation by low-frequency stimulation at hippocampal CA1 synapses. *J. Neurosci.* 19 9728–9738. 10.1523/jneurosci.19-22-09728.1999 10559382 PMC6782980

[B27] Huang C. C. Liang Y. C. Hsu K. S. (2001). Characterization of the mechanism underlying the reversal of long term potentiation by low frequency stimulation at hippocampal CA1 synapses. *J. Biol. Chem.* 276 48108–48117. 10.1074/jbc.M106388200 11679581

[B28] HzFroc D. J. Chapman C. A. Trepel C. Racine R. J. (2000). Long-term depression and depotentiation in the sensorimotor cortex of the freely moving rat. *J. Neurosci.* 20 438–445. 10.1523/jneurosci.20-01-00438.2000 10627619 PMC6774135

[B29] Kang-Park M.-H. Sarda M. A. Jones K. H. Moore S. D. Shenolikar S. Clark S. (2003). Protein phosphatases mediate depotentiation induced by high-intensity theta-burst stimulation. *J. Neurophysiol.* 89 684–690. 10.1152/jn.01041.2001 12574446

[B30] Kim W. B. Cho J. H. (2017). Encoding of discriminative fear memory by input-specific LTP in the amygdala. *Neuron* 95 1129–1146.e5. 10.1016/j.neuron.2017.08.004 28823727

[B31] Kitamura T. Saitoh Y. Takashima N. Murayama A. Niibori Y. Ageta H. (2009). Adult neurogenesis modulates the hippocampus-dependent period of associative fear memory. *Cell* 139 814–827. 10.1016/j.cell.2009.10.020 19914173

[B32] Kramár E. A. Lynch G. (2003). Developmental and regional differences in the consolidation of long-term potentiation. *Neuroscience* 118 387–398. 10.1016/s0306-4522(02)00916-8 12699775

[B33] Kuhn H. G. Dickinson-Anson H. Gage F. H. (1996). Neurogenesis in the dentate gyrus of the adult rat: age-related decrease of neuronal progenitor proliferation. *J. Neurosci.* 16 2027–2033. 10.1523/jneurosci.16-06-02027.1996 8604047 PMC6578509

[B34] Laplagne D. A. Esposito M. S. Piatti V. C. Morgenstern N. A. Zhao C. van Praag H. (2006). Functional convergence of neurons generated in the developing and adult hippocampus. *PLoS Biol.* 4:e409. 10.1371/journal.pbio.0040409 17121455 PMC1637132

[B35] Larson J. Lynch G. (1986). Induction of synaptic potentiation in hippocampus by patterned stimulation involves two events. *Science* 232 985–988. 10.1126/science.3704635 3704635

[B36] Lee H. K. Barbarosie M. Kameyama K. Bear M. F. Huganir R. L. (2000). Regulation of distinct AMPA receptor phosphorylation sites during bidirectional synaptic plasticity. *Nature* 405 955–959. 10.1038/35016089 10879537

[B37] Lee H. K. Kameyama K. Huganir R. L. Bear M. F. (1998). NMDA induces long-term synaptic depression and dephosphorylation of the GluR1 subunit of AMPA receptors in hippocampus. *Neuron* 21 1151–1162. 10.1016/s0896-6273(00)80632-7 9856470

[B38] Lewis D. A. Levitt P. (2002). Schizophrenia as a disorder of neurodevelopment. *Annu. Rev. Neurosci.* 25 409–432. 10.1146/annurev.neuro.25.112701.142754 12052915

[B39] Liu L. Wong T. P. Pozza M. F. Lingenhoehl K. Wang Y. Sheng M. (2004). Role of NMDA receptor subtypes in governing the direction of hippocampal synaptic plasticity. *Science* 304 1021–1024. 10.1126/science.1096615 15143284

[B40] Liu X. B. Murray K. D. Jones E. G. (2004). Switching of NMDA receptor 2A and 2B subunits at thalamic and cortical synapses during early postnatal development. *J. Neurosci.* 24 8885–8895. 10.1523/JNEUROSCI.2476-04.2004 15470155 PMC6729956

[B41] Loftis J. M. Janowsky A. (2003). The N-methyl-D-aspartate receptor subunit NR2B: localization, functional properties, regulation, and clinical implications. *Pharmacol. Ther.* 97 55–85. 10.1016/s0163-7258(02)00302-9 12493535

[B42] Lynch M. A. (2004). Long-term potentiation and memory. *Physiol. Rev.* 84 87–136. 10.1152/physrev.00014.2003 14715912

[B43] Malenka R. C. (2003). The long-term potential of LTP. *Nat. Rev. Neurosci.* 4 923–926. 10.1038/nrn1258 14595403

[B44] Malenka R. C. Bear M. F. (2004). LTP and LTD: an embarrassment of riches. *Neuron* 44 5–21. 10.1016/j.neuron.2004.09.012 15450156

[B45] Maletic-Savatic M. Malinow R. Svoboda K. (1999). Rapid dendritic morphogenesis in CA1 hippocampal dendrites induced by synaptic activity. *Science* 283 1923–1927. 10.1126/science.283.5409.1923 10082466

[B46] Mao Y. Ge X. Frank C. L. Madison J. M. Koehler A. N. Doud M. K. (2009). Disrupted in schizophrenia 1 regulates neuronal progenitor proliferation via modulation of GSK3beta/beta-catenin signaling. *Cell* 136 1017–1031. 10.1016/j.cell.2008.12.044 19303846 PMC2704382

[B47] Marin-Burgin A. Mongiat L. A. Pardi M. B. Schinder A. F. (2012). Unique processing during a period of high excitation/inhibition balance in adult-born neurons. *Science* 335 1238–1242. 10.1126/science.1214956 22282476 PMC3385415

[B48] Martin S. J. Grimwood P. D. Morris R. G. M. (2000). Synaptic plasticity and memory: an evaluation of the hypothesis. *Annu. Rev. Neurosci.* 23 649–711. 10.1146/annurev.neuro.23.1.649 10845078

[B49] Massa F. Koehl M. Wiesner T. Grosjean N. Revest J. M. Piazza P. V. (2011). Conditional reduction of adult neurogenesis impairs bidirectional hippocampal synaptic plasticity. *Proc. Natl. Acad. Sci. U.S.A.* 108 6644–6649. 10.1073/pnas.1016928108 21464314 PMC3081011

[B50] Massey P. V. Johnson B. E. Moult P. R. Auberson Y. P. Brown M. W. Molnar E. (2004). Differential roles of NR2A and NR2B-containing NMDA receptors in cortical long-term potentiation and long-term depression. *J. Neurosci.* 24 7821–7828. 10.1523/JNEUROSCI.1697-04.2004 15356193 PMC6729941

[B51] Monyer H. Burnashev N. Laurie D. J. Sakmann B. Seeburg P. H. (1994). Developmental and regional expression in the rat brain and functional properties of four NMDA receptors. *Neuron* 12 529–540. 10.1016/0896-6273(94)90210-0 7512349

[B52] Monyer H. Sprengel R. Schoepfer R. Herb A. Higuchi M. Lomeli H. (1992). Heteromeric NMDA receptors: molecular and functional distinction of subtypes. *Science* 256 1217–1221. 10.1126/science.256.5060.1217 1350383

[B53] Mu Y. Zhao C. Gage F. H. (2011). Dopaminergic modulation of cortical inputs during maturation of adult-born dentate granule cells. *J. Neurosci.* 31 4113–4123. 10.1523/JNEUROSCI.4913-10.2011 21411652 PMC3073019

[B54] Mu Y. Zhao C. Toni N. Yao J. Gage F. H. (2015). Distinct roles of NMDA receptors at different stages of granule cell development in the adult brain. *eLife* 4:e07871. 10.7554/eLife.07871 26473971 PMC4608052

[B55] Muller D. Hefft S. Figurov A. (1995). Heterosynaptic interactions between LTP and LTD in CA1 hippocampal slices. *Neuron* 14 599–605. 10.1016/0896-6273(95)90316-x 7695906

[B56] Nabavi S. Fox R. Proulx C. D. Lin J. Y. Tsien R. Y. Malinow R. (2014). Engineering a memory with LTD and LTP. *Nature* 511 348–352. 10.1038/nature13294 24896183 PMC4210354

[B57] Nakashiba T. Cushman J. D. Pelkey K. A. Renaudineau S. Buhl D. L. McHugh T. J. (2012). Young dentate granule cells mediate pattern separation, whereas old granule cells facilitate pattern completion. *Cell* 149 188–201. 10.1016/j.cell.2012.01.046 22365813 PMC3319279

[B58] Nguyen P. Abel T. Kandel E. (1994). Requirement of a critical period of transcription for induction of a late phase of LTP. *Science* 265 1104–1107. 10.1126/science.8066450 8066450

[B59] O’Dell T. J. Kandel E. R. (1994). Low-frequency stimulation erases LTP through an NMDA receptor-mediated activation of protein phosphatases. *Learn. Mem.* 1 129–139. 10467591

[B60] Ouchi Y. Banno Y. Shimizu Y. Ando S. Hasegawa H. Adachi K. (2013). Reduced adult hippocampal neurogenesis and working memory deficits in the Dgcr8-deficient mouse model of 22q11.2 deletion-associated schizophrenia can be rescued by IGF2. *J. Neurosci.* 33 9408–9419. 10.1523/JNEUROSCI.2700-12.2013 23719809 PMC6618567

[B61] Paoletti P. Bellone C. Zhou Q. (2013). NMDA receptor subunit diversity: impact on receptor properties, synaptic plasticity and disease. *Nat. Rev. Neurosci.* 14 383–400. 10.1038/nrn3504 23686171

[B62] Philpot B. D. Cho K. K. Bear M. F. (2007). Obligatory role of NR2A for metaplasticity in visual cortex. *Neuron* 53 495–502. 10.1016/j.neuron.2007.01.027 17296552 PMC1847797

[B63] Pilz G. A. Bottes S. Betizeau M. Jorg D. J. Carta S. Simons B. D. (2018). Live imaging of neurogenesis in the adult mouse hippocampus. *Science* 359 658–662. 10.1126/science.aao5056 29439238 PMC6986926

[B64] Qi Y. Hu N. W. Rowan M. J. (2013). Switching off LTP: mGlu and NMDA receptor–dependent novelty exploration–induced depotentiation in the rat hippocampus. *Cereb. Cortex* 23 932–939. 10.1093/cercor/bhs086 22490551

[B65] Quinlan E. M. Lebel D. Brosh I. Barkai E. (2004). A molecular mechanism for stabilization of learning-induced synaptic modifications. *Neuron* 41 185–192. 10.1016/s0896-6273(03)00874-2 14741100

[B66] Reif A. Fritzen S. Finger M. Strobel A. Lauer M. Schmitt A. (2006). Neural stem cell proliferation is decreased in schizophrenia, but not in depression. *Mol. Psychiatry* 11 514–522. 10.1038/sj.mp.4001791 16415915

[B67] Roberts E. B. Ramoa A. S. (1999). Enhanced NR2A subunit expression and decreased NMDA receptor decay time at the onset of ocular dominance plasticity in the ferret. *J. Neurophysiol.* 81 2587–2591. 10.1152/jn.1999.81.5.2587 10322092

[B68] Rumbaugh G. Vicini S. (1999). Distinct synaptic and extrasynaptic NMDA receptors in developing cerebellar granule neurons. *J. Neurosci.* 19 10603–10610. 10.1523/jneurosci.19-24-10603.1999 10594044 PMC6784938

[B69] Schinder A. F. Gage F. H. (2004). A hypothesis about the role of adult neurogenesis in hippocampal function. *Physiology (Bethesda)* 19 253–261. 10.1152/physiol.00012.2004 15381753

[B70] Schmidt-Hieber C. Jonas P. Bischofberger J. (2004). Enhanced synaptic plasticity in newly generated granule cells of the adult hippocampus. *Nature* 429 184–187. 10.1038/nature02553 15107864

[B71] Shamir A. Kwon O. B. Karavanova I. Vullhorst D. Leiva-Salcedo E. Janssen M. J. (2012). The importance of the NRG-1/ErbB4 pathway for synaptic plasticity and behaviors associated with psychiatric disorders. *J. Neurosci.* 32 2988–2997. 10.1523/JNEUROSCI.1899-11.2012 22378872 PMC3302154

[B72] Sheng M. Cummings J. Roldan L. A. Jan Y. N. Jan L. Y. (1994). Changing subunit composition of heteromeric NMDA receptors during development of rat cortex. *Nature* 368 144–147. 10.1038/368144a0 8139656

[B73] Skaggs W. E. McNaughton B. L. Wilson M. A. Barnes C. A. (1996). Theta phase precession in hippocampal neuronal populations and the compression of temporal sequences. *Hippocampus* 6 149–172. 10.1002/(SICI)1098-10631996 8797016

[B74] Song Y. Zhang M. Tao X. Xu Z. Zhang L. Zheng Y. (2016). A single-cell-type real-time pcr method based on a modified patch-pipette cell harvesting system. *Mol. Biotechnol.* 58 558–565. 10.1007/s12033-016-9953-y 27271017

[B75] Staubli U. Scafidi J. (1999). Time-dependent reversal of long-term potentiation in area CA1 of the freely moving rat induced by theta pulse stimulation. *J. Neurosci.* 19 8712–8719. 10.1523/jneurosci.19-19-08712.1999 10493772 PMC6783049

[B76] Sugita M. Yamazaki Y. Goto J. I. Fujiwara H. Aihara T. Mikoshiba K. (2016). Role of postsynaptic inositol 1, 4, 5-trisphosphate receptors in depotentiation in guinea pig hippocampal CA1 neurons. *Brain Res.* 1642 154–162. 10.1016/j.brainres.2016.03.033 27018292

[B77] Takahashi T. Feldmeyer D. Suzuki N. Onodera K. Cull-Candy S. G. Sakimura K. (1996). Functional correlation of NMDA receptor epsilon subunits expression with the properties of single-channel and synaptic currents in the developing cerebellum. *J. Neurosci.* 16 4376–4382. 10.1523/jneurosci.16-14-04376.1996 8699248 PMC6578868

[B78] Tsai T. C. Huang C. C. Hsu K. S. (2018). Infantile amnesia is related to developmental immaturity of the maintenance mechanisms for long-term potentiation. *Mol. Neurobiol.* 56 907–919. 10.1007/s12035-018-1119-4 29804230

[B79] Vallano M. L. Lambolez B. Audinat E. Rossier J. (1996). Neuronal activity differentially regulates NMDA receptor subunit expression in cerebellar granule cells. *J. Neurosci.* 16 631–639. 10.1523/jneurosci.16-02-00631.1996 8551347 PMC6578662

[B80] van Praag H. Schinder A. F. Christie B. R. Toni N. Palmer T. D. Gage F. H. (2002). Functional neurogenesis in the adult hippocampus. *Nature* 415 1030–1034. 10.1038/4151030a 11875571 PMC9284568

[B81] Vicini S. Wang J. F. Li J. H. Zhu W. J. Wang Y. H. Luo J. H. (1998). Functional and pharmacological differences between recombinant N-methyl-D-aspartate receptors. *J. Neurophysiol.* 79 555–566. 10.1152/jn.1998.79.2.555 9463421

[B82] Wagner J. J. Alger B. E. (1995). GABAergic and developmental influences on homosynaptic LTD and depotentiation in rat hippocampus. *J. Neurosci.* 15 1577–1586. 10.1523/jneurosci.15-02-01577.1995 7869119 PMC6577799

[B83] Wagner J. J. Alger B. E. (1996). Homosynaptic LTD and depotentiation: do they differ in name only? *Hippocampus* 6 24–29. 10.1002/(SICI)1098-10631996 8878738

[B84] Wang C. C. Held R. G. Chang S. C. Yang L. Delpire E. Ghosh A. (2011). A critical role for GluN2B-containing NMDA receptors in cortical development and function. *Neuron* 72 789–805. 10.1016/j.neuron.2011.09.023 22153375

[B85] Wenzel A. Fritschy J. M. Mohler H. Benke D. (1997). NMDA receptor heterogeneity during postnatal development of the rat brain: differential expression of the NR2A, NR2B, and NR2C subunit proteins. *J. Neurochem.* 68 469–478. 10.1046/j.1471-4159.1997.68020469.x 9003031

[B86] Wixted J. T. (2004). The psychology and neuroscience of forgetting. *Annu. Rev. Psychol.* 55 235–269. 10.1146/annurev.psych.55.090902.141555 14744216

[B87] Xu L. Anwyl R. Rowan M. J. (1998). Spatial exploration induces a persistent reversal of long-term potentiation in rat hippocampus. *Nature* 394 891–894. 10.1038/29783 9732871

[B88] Yang S. N. Tang Y. G. Zucker R. S. (1999). Selective induction of LTP and LTD by postsynaptic [Ca2+]i elevation. *J. Neurophysiol.* 81 781–787. 10.1152/jn.1999.81.2.781 10036277

[B89] Zhang L. I. Poo M. M. (2001). Electrical activity and development of neural circuits. *Nat. Neurosci.* 4(Suppl.), 1207–1214. 10.1038/nn753 11687831

[B90] Zhao C. Teng E. M. Summers R. G. Jr. Ming G. L. Gage F. H. (2006). Distinct morphological stages of dentate granule neuron maturation in the adult mouse hippocampus. *J. Neurosci.* 26 3–11. 10.1523/JNEUROSCI.3648-05.2006 16399667 PMC6674324

[B91] Zhou Q. Poo M. M. (2004). Reversal and consolidation of activity-induced synaptic modification. *Trends Neurosci.* 27 378–383. 10.1016/j.tins.2004.05.006 15219736

[B92] Zhou Q. Tao H. W. Poo M. M. (2003). Reversal and stabilization of synaptic modifications in a developing visual system. *Science* 300 1953–1957. 10.1126/science.1082212 12817152

[B93] Zhu Y. Pak D. Qin Y. McCormack S. G. Kim M. J. Baumgart J. P. (2005). Rap2-JNK removes synaptic AMPA receptors during depotentiation. *Neuron* 46 905–916. 10.1016/j.neuron.2005.04.037 15953419

