# Supplementary material for: Development of Depotentiation in Adult-Born Dentate Granule Cells
Source: Front Cell Dev Biol. 2019 Oct 16;7:236. doi: 10.3389/fcell.2019.00236 (PMC6805727; doi:10.3389/fcell.2019.00236)
Supplement: Supplementary file 1 [file Table_1.DOCX]

**Development of depotentiation in adult-born dentate granule cells**

***Supplementary Material***


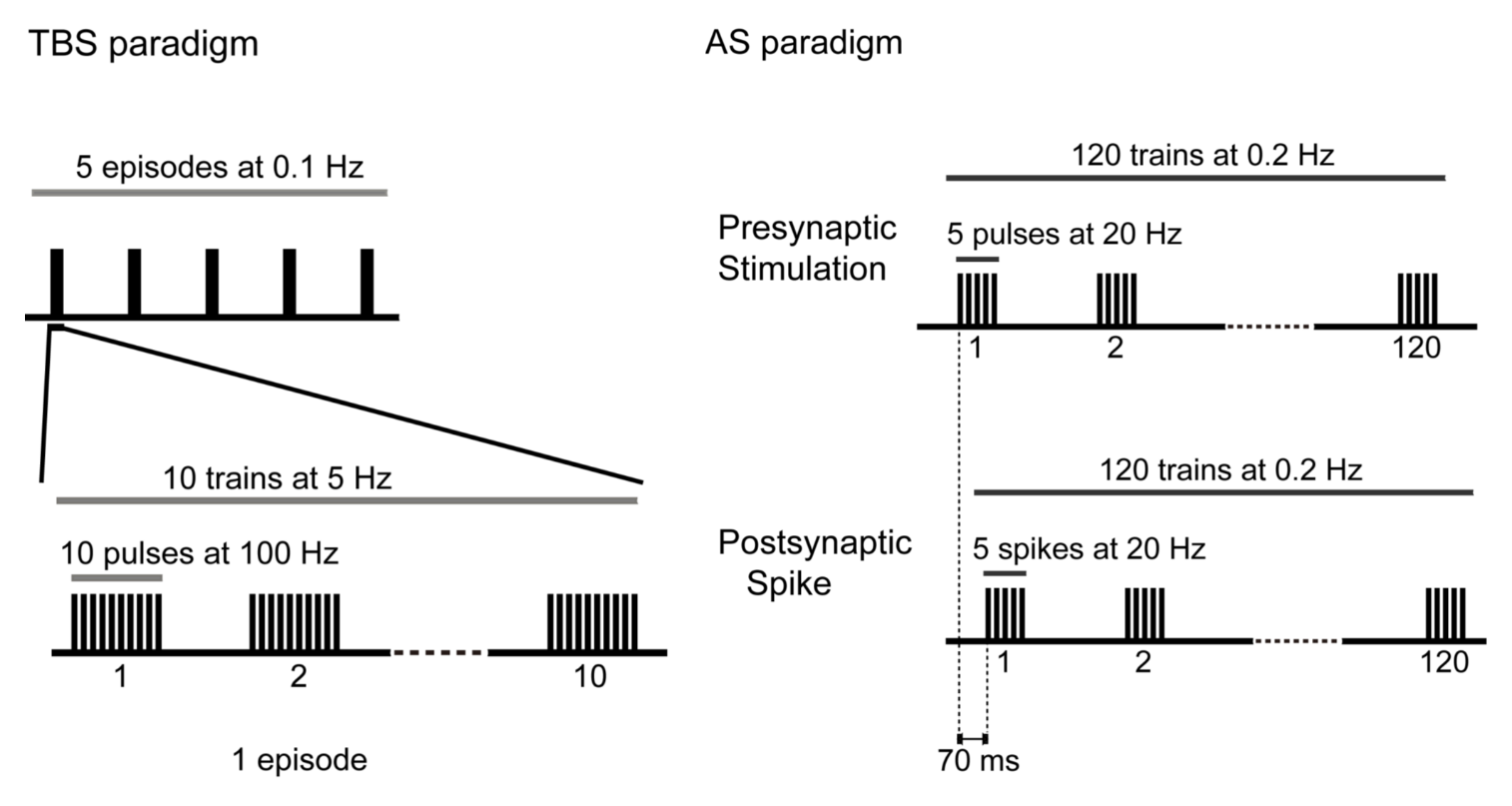
**Figure S1.** TBS and AS paradigm. Left, TBS paradigm, five episodes of TBS applied at 0.1 Hz. Each episode of TBS consisted of 10 trains of stimuli delivered every 200 ms, with ten pulses at 100 Hz in each train. Right, AS paradigm, a combination of presynaptic stimulation and postsynaptic spikes, in which the presynaptic stimulation was 70 ms anterior to the postsynaptic spikes. The presynaptic stimulation and the postsynaptic spikes both consisted of 120 trains at 0.2 Hz, and each train contained 5 pulses at 20 Hz.

**
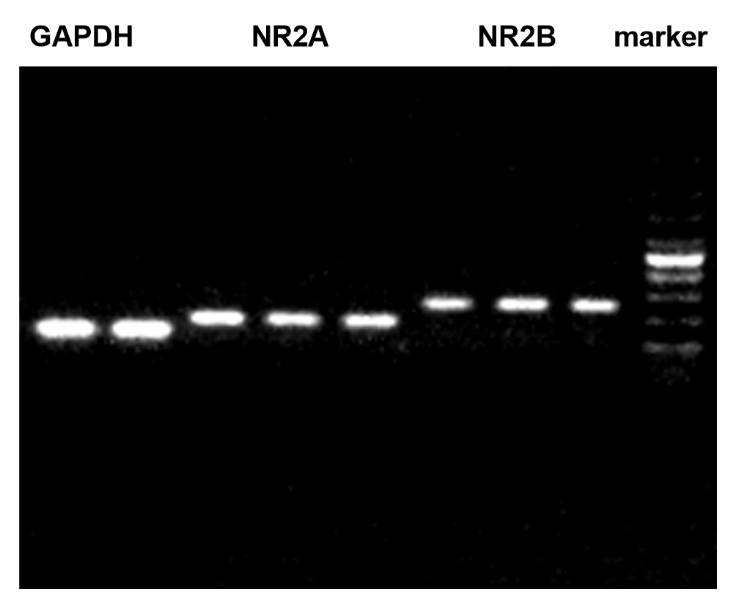
**

**Figure S2.** An electrophoresis image obtained by using the gel imaging analysis system to verify the products of NR2A, NR2B and GAPDH after PCR. The size of the marker strip as follows (from bottom to top): 100bp, 200bp, 300bp, 400bp, 500bp.
